# Post-bariatric Hypoglycemia Management: A Gulf Cooperation Council Consensus Statement

**DOI:** 10.1210/jendso/bvaf225

**Published:** 2025-12-30

**Authors:** Elamin Abdelgadir, Fauzia Rashid, Fatheya Al Awadi, Noor Badar Al Busaidi, Nasreen Alfaris, Mohammed Al Hadad, Juma Alkaabi, Amira Al Kharusi, Ebaa Al Ozairi, Dalal Alromaihi, Aseel AlSaleh, Alaaeldin Bashier, Rahila Bhatti, Wahiba Elhag, Carel W le Roux, Sara G I Suliman

**Affiliations:** Mohamed Bin Rashid University, Dubai 505055, UAE; Endocrine Department, Dubai Hospital, Dubai 7272, UAE; Mohamed Bin Rashid University, Dubai 505055, UAE; Endocrine Department, Dubai Hospital, Dubai 7272, UAE; Mohamed Bin Rashid University, Dubai 505055, UAE; Endocrine Department, Dubai Hospital, Dubai 7272, UAE; National Diabetes and Endocrine Center, Royal Hospital, Muscat 111, Sultanate of Oman; Department of Endocrinology, King Fahad Medical City, Riyadh 12231, Saudi Arabia; Department of Bariatric & Metabolic Surgery, Healthpoint Hospital, Abu Dhabi M42, UAE; Internal Medicine Department, College of Medicine and Health Sciences, CMHS UAEU, Al Ain 1551, UAE; National Diabetes and Endocrine Center, Royal Hospital, Muscat 111, Sultanate of Oman; Department of Endocrinology, Dasman Diabetes Institute, Dasman 15462, Kuwait; Awali Hospital, Royal College of Surgeons in Ireland, Awali 973, Bahrain; The Department of Family and Community Medicine, Arabian Gulf University, Manama 26671, Kingdom of Bahrain; Mohamed Bin Rashid University, Dubai 505055, UAE; Endocrine Department, Dubai Hospital, Dubai 7272, UAE; Department of Endocrinology, Genesis Healthcare Center, Dubai Science Park Towers, Dubai, UAE; National Bariatric Center, Qatar Metabolic Institute, Hamad Medical Corporation, Weill Cornell Medicine—Qatar, Doha 3050, Qatar; Department of Medicine, University College Dublin Diabetes Complications Research Centre, University College Dublin, P.O Box 1, Dublin, Ireland; Department of Endocrinology, Imperial College London Diabetes Centre (ICLDC), Abu Dhabi 48338, UAE

**Keywords:** obesity, metabolic and bariatric surgery, post-bariatric hypoglycemia, Gulf region, RYGB, dumping syndrome, postprandial, PBH, GCC, bariatric surgery

## Abstract

Post-bariatric hypoglycemia (PBH) is a late complication of metabolic and bariatric surgery that typically manifests over 1 year after the procedure. The clinical manifestation spans from mild hypoglycemia responsive to dietary modifications to severe hypoglycemia with neuroglycopenic symptoms. Despite its clinical significance and the growing body of evidence, the management of PBH remains heterogeneous, primarily due to its complex, multifactorial pathophysiology, the lack of standardized diagnostic criteria and Food and Drug Administration (FDA)-approved pharmacological treatments, and discrepancies in diagnostic and therapeutic approaches across available clinical guidelines. This consensus aims to establish a unified, evidence-based, and patient-centered management protocol for PBH.

A panel of 16 experts, encompassing representatives from all Gulf Cooperation Council (GCC) countries and Europe, conducted an extensive review of the current literature to assemble the most recent evidence on PBH management. The panel then collaboratively developed a set of statements to standardize the diagnosis and treatment of PBH. Consensus was reached on all the statements using the Delphi method.

Consensus was attained on 45 statements encompassing the entire PBH management continuum, including diagnosis, dietary management, patient education, and pharmacological treatment, with special considerations during pregnancy, long-term monitoring, and practical aspects of clinical management.

Implementing these consensus statements into clinical practice will contribute to the standardization of PBH management. Furthermore, the statements highlight significant gaps in PBH management, including the lack of PBH-specific therapies and the scarcity of robust trials, which urgently require attention in future research and clinical development.

Study importance questions **What is already known?**

- The global prevalence of obesity, now recognized as a chronic, relapsing disease, is on the rise, with countries in the Gulf region witnessing a much steeper increase.
- Given the significant benefits of metabolic and bariatric surgery (MBS) in the form of durable weight loss and improvements in obesity-related comorbidities and metabolic syndrome, its eligibility criteria have expanded compared to the past.
- However, MBS is associated with late complications such as post-bariatric hypoglycemia (PBH).
- Despite its clinical significance, there is significant variability in PBH management.

**What does this paper add?**

- This paper provides comprehensive and consensus-based guidance on PBH management that is evidence-based and patient-centric.

**How might these results change the direction of research or the focus of clinical practice?**

- The clinical significance of PBH highlighted in this study may raise awareness about this post-bariatric complication among healthcare professionals.
- The guidance provided in this paper would provide physicians in the Gulf region with standardized, evidence-based strategies and practical insights to optimize PBH management and improve patient outcomes.
- The unmet needs highlighted in this study may encourage the development of PBH-specific therapies and drive research efforts to better understand its pathophysiology.

## Obesity in the Gulf Cooperation Council: A Growing Concern

In recent decades, obesity rates have surged, with prevalence doubling from 1990 to 2022 [1]. While obesity rates have increased worldwide, the Middle East and North Africa (MENA) region, with an estimated population of over 450 million, has experienced an even sharper rise [2-4]. The pooled obesity prevalence in Middle Eastern countries is estimated at 21.17%, with rates ranging from nearly 40% in Syria to 8.8% in Yemen [5]. Furthermore, the Gulf Cooperation Council (GCC) countries, a subset of MENA, hold the highest obesity rates globally. Prevalence estimates in the United Arab Emirates (UAE), Saudi Arabia, Qatar, and Kuwait range from 32% to 38%, significantly exceeding the global average [6].

Apart from adversely impacting the quality of life (QoL), obesity, a complex, chronic, relapsing disease characterized by increased fat mass, is associated with various health conditions, including cardiovascular disease and type 2 diabetes, as well as several other diseases, such as cancer, osteoarthritis, liver and kidney disease, sleep apnea, and depression [7, 8]. According to the International Diabetes Federation (IDF) data, 1 in every 6 adults in the MENA region lives with diabetes, compared to about 1 in 10 adults globally [9]. These grim statistics underscore the impact of obesity in the MENA region and the consequent surge in the number of bariatric surgeries performed to manage obesity and its associated complications over the last 2 or 3 decades [10, 11].

## Metabolic and Bariatric Surgery in the Treatment of Obesity: Current Trends and Challenges

Metabolic and bariatric surgery (MBS) has emerged as an effective obesity treatment. It facilitates weight loss and improves QoL as well as numerous obesity-linked complications. The most commonly performed bariatric surgery procedures are sleeve gastrectomy (SG) and Roux-en-Y gastric bypass (RYGB) [12]. According to estimates from the American Society for Metabolic and Bariatric Surgery (ASMBS), the number of bariatric surgeries performed in the United States rose from 158 000 in 2011 to 279 967 in 2022, a 77% increase in just 11 years. In 2022, nearly 80% of these procedures were SG and RYGB [13]. Other types of MBS include one-anastomosis gastric bypass (OAGB), biliopancreatic diversion with duodenal switch (BPD-DS), and single anastomosis duodenal-ileal bypass with sleeve gastrectomy (SADI-S) [14].

The 1991 National Institutes of Health (NIH) Consensus Statement recommended bariatric surgery for patients with a body mass index (BMI) ≥40 kg/m^2^ without obesity-related complications or a BMI ≥35 kg/m^2^ with one or more obesity-related complications, provided the procedure did not pose excessive risk [8, 15]. Healthcare practitioners have applied these criteria until recently to select surgery candidates. However, emerging long-term data on obesity and the effectiveness of bariatric surgery have prompted a reevaluation of this recommendation. Thus, the 2022 joint guidelines published by the ASMBS and International Federation for the Surgery of Obesity and Metabolic Disorders (IFSO) have extended the eligibility criteria for bariatric surgery, recommending it for individuals with a BMI ≥35 kg/m^2^ (class II and higher), irrespective of the presence or severity of any complications, and for type 2 diabetes patients with a BMI ≥30 kg/m^2^ (class I and higher). It may also be considered in those with a BMI between 30 and 34.9 kg/m^2^ (class I obesity) who do not show favorable weight loss with nonsurgical interventions [16].

**Figure 1.**
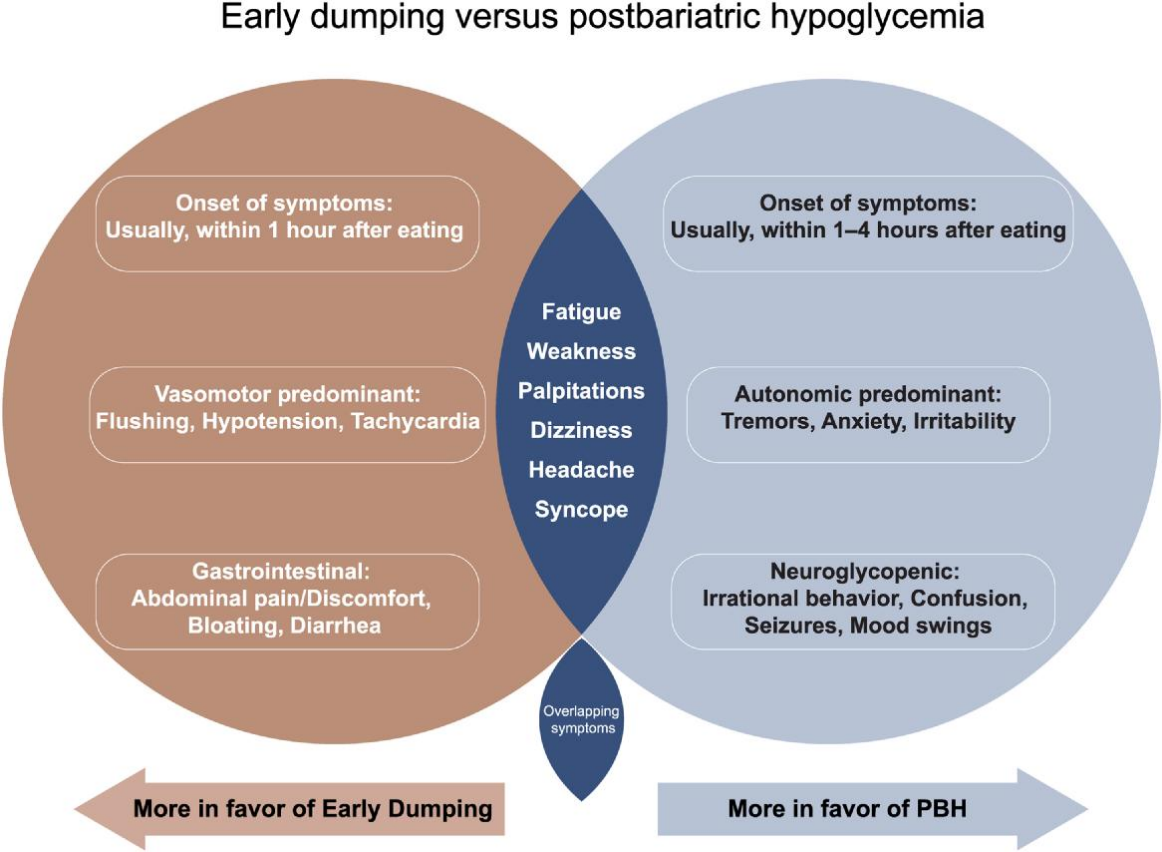
Early dumping vs post-bariatric hypoglycemia. Abbreviation: GI, gastrointestinal.

While an effective weight loss and metabolic intervention, MBS can lead to multiple short- and long-term postoperative complications and nutritional deficiencies [17, 18]. Additionally, physicians must remain vigilant for complications such as post-bariatric hypoglycemia (PBH) that can develop years after the surgery. PBH typically presents more than a year after surgery. It is characterized by postprandial hyperinsulinemic hypoglycemia with autonomic or neuroglycopenic symptoms [19-21]. PBH presents in a spectrum of severity, ranging from mild hypoglycemic episodes alleviated by dietary modifications to severe hypoglycemia with accompanying neuroglycopenic symptoms [19, 20, 22]. It has also been historically referred to in the literature as the “late” dumping syndrome to distinguish it from the “early” dumping syndrome that develops within an hour of ingesting food and triggers gastrointestinal and vasomotor symptoms without hypoglycemia (see Fig. 1) [23-25]. However, there is debate over whether it is appropriate to refer to PBH as late dumping syndrome, given that its pathophysiology extends beyond the typical dumping mechanisms involving rapid food transit from the stomach to the small intestine. Thus, according to some researchers, referring to PBH as late dumping syndrome might be a misnomer [26]. On the other hand, some experts argue that the proposed pathophysiology of PBH, although distinct, overlaps with the dumping mechanism that subsequently triggers excessive insulin and incretin release, contributing to hypoglycemia. Hence, the terminology late dumping syndrome may be appropriate in this context [27].

## Pathophysiology of Post-Bariatric Hypoglycemia: Multifactorial, Heterogeneous, and Still Unclear

**Figure 2.**
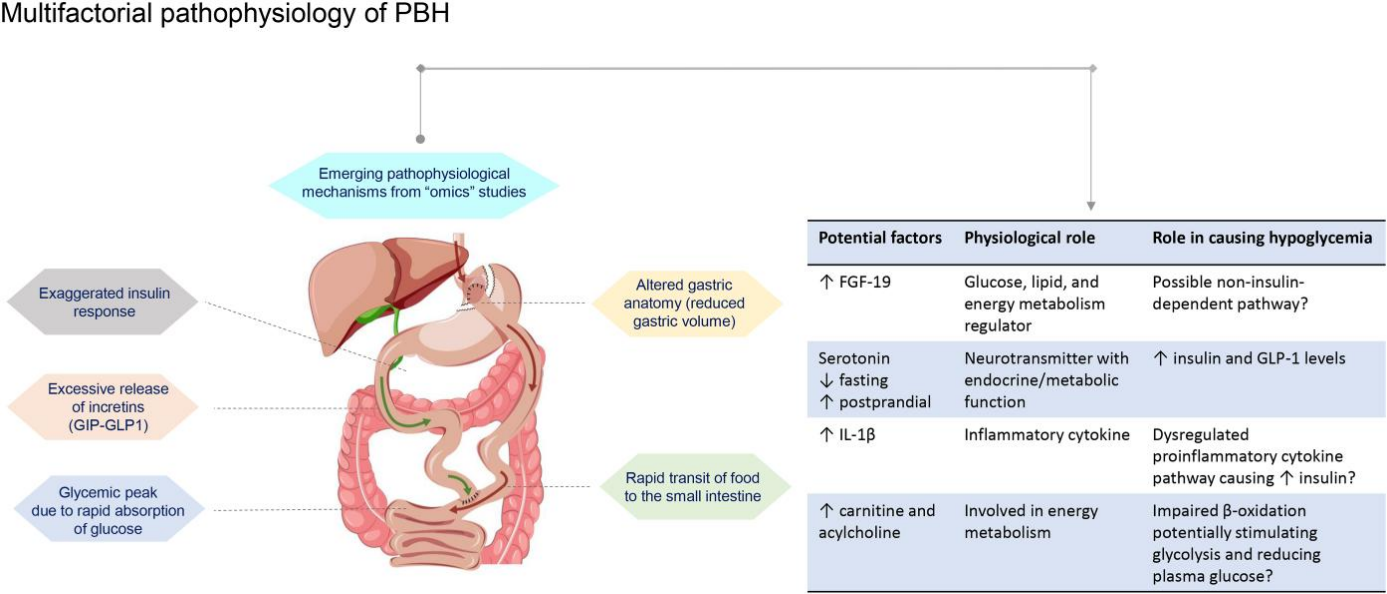
Multifactorial pathophysiology of PBH. Abbreviations: GIP, gastric inhibitory polypeptide; GLP-1, glucagon-like peptide 1; FGF, fibroblast growth factor; IL, interleukin.

Several hormonal-mechanical hypotheses have been put forth to explain the pathophysiology of PBH (see Fig. 2) [28, 29]. Some commonly proposed mechanisms revolve around (i) accelerated food transit; (ii) altered hormonal activity and counterregulatory responses; and (iii) intestinal adaptation that develops over time in response to postoperative changes in gastrointestinal physiology.

Postoperative anatomical changes accelerate food transit into the small intestine, upregulating glucose transporters and causing postprandial hyperglycemia. This glucose peak is followed by an exaggerated glucagon-like peptide 1 (GLP-1)- stimulated, glucose-dependent insulin release and, consequently, hypoglycemia. Studies also show an inadequate suppression of basal insulin secretion by β-cells in response to hypoglycemia, which can contribute to PBH [23, 28, 30, 31]. Thus, a disruption in insulin regulation in response to both hyperglycemia and hypoglycemia may be one of the several pathways that play a role in PBH pathophysiology.

Counterregulatory hormones, such as glucagon, maintain glucose homeostasis when glucose levels fall below a certain threshold. However, these thresholds are lowered in those with recurrent hypoglycemia [32]. In this context, it should be noted that while the counterregulatory glucagon response to postprandial hypoglycemia is blunted in individuals following MBS compared to nonsurgical controls, no difference is seen between post-bariatric patients with and without PBH, irrespective of the type of surgery [33]. The evaluation of gut and pancreatic hormone dynamics by Lobato et al highlights the complex interplay underlying PBH. The study showed that postprandial hyperinsulinemia was an essential component of PBH. Also, an early glucagon excursion (15 minutes after the meal) was protective against hypoglycemia, possibly explaining the individual variability in glycemic outcomes. Impairment of this early response may also contribute to PBH [34]. The pathophysiological mechanism of PBH driven by these hormonal interactions is complex and is gradually being elucidated.

The manifestation of PBH symptoms >1 year after bariatric surgery suggests that the underlying mechanisms must involve changes in gastrointestinal structure, function, and hormonal regulation that occur gradually over time [35]. The rapid transit of food and altered pH of contents entering the intestine due to surgical modifications have been thought to promote “enteroplasticity,” that is, intestinal adaptation. These adaptations have been hypothesized to occur via changes in: (i) gastrointestinal morphology (cell-structural and morphological changes); (ii) neuronal activity/innervation; (iii) enteroendocrine changes (altered gut hormones, increased enteroendocrine cell numbers, increased sensitivity to stimuli); and (iv) altered intestinal nutrient sensing/signaling (changes in nutrient transporters, processing, byproducts, and intracellular signaling pathways) [36]. However, more research is needed to delineate these adaptations’ precise nature and extent and the molecular pathways involved.

While several hypotheses have been formulated to explain the pathophysiological processes leading to PBH, the precise mechanism, which likely involves a convergence of these multiple pathways, remains to be elucidated.

## Emerging Evidence on PBH Pathophysiology from -Omics Studies

Individual genetic differences are also likely to impact the hormonal responses, regulatory and counterregulatory pathways, and intestinal adaptations. These differences could help explain why some individuals are more susceptible to complications like PBH than others [35]. Emerging metabolomic and proteomic studies are beginning to uncover the molecular mechanisms underlying PBH pathophysiology (see Fig. 2) [37-40].

An analysis of the plasma metabolome and proteome during fasting and postprandial (after a mixed meal) periods in 3 groups—those with post-RYGB PBH, those who were asymptomatic post-RYGB, and those with obesity or who were overweight (nonsurgical controls)—has revealed distinct “metabolic signatures” in the 3 groups and identified increased fibroblast growth factor (FGF)-19 levels, and decreased fasting and increased postprandial serotonin levels in the PBH cohort [37, 39]. Animal studies suggest that serotonin administration can elevate insulin and GLP-1 levels, leading to a reduction in plasma glucose, and implying a role for serotonin in the development of PBH and as a potential therapeutic target in the future [37]. FGF-19, through FXR (a bile acid-activated receptor), regulates glucose, lipid, and energy metabolism. Elevated levels of FGF-19 can have implications for postprandial metabolic responses and a potential role in PBH pathophysiology. Another metabolomics analysis by Aydin et al found differences in carnitine and acylcholines, key metabolites involved in energy metabolism, in PBH patients compared to non-PBH patients. This study also reported increased levels of FGF-19 and FGF-21 following bariatric surgery; however, their levels were similar between PBH and non-PBH patients [38].

An exploratory gene expression analysis found that inflammatory cytokines like interleukin (IL-)1β and IL-6 were upregulated in monocytes of patients during hypoglycemia events after a mixed meal test. This finding is significant in PBH, as glucose-driven IL-1β can induce insulin secretion. The same study also showed that empagliflozin, a sodium-glucose cotransporter (SGLT)-2 inhibitor via a reduction in postprandial hyperglycemia peak, and anakinra, an IL-1 receptor antagonist, via IL-1 signaling blockade, could reduce hypoglycemia in at-risk patients after gastric bypass surgery [29].

While these studies are just the foundation for a new era in understanding PBH, they do not yet clarify whether the observed molecular changes are causative or secondary to hypoglycemia and the exaggerated postprandial response in PBH [39]. We hope that more -omics studies in the future will provide comprehensive insights into the complex molecular mechanisms underlying individual variations in response to bariatric surgery, including the development of PBH [41].

## Prevalence and Risk Factors of PBH

Variable estimations of PBH prevalence have been reported in the literature primarily because of the different criteria used to diagnose it. Reported prevalence estimates range from 19% to 75% in studies using continuous glucose monitoring (CGM) and provocation tests and from 0.1% to 0.9% in registry-based studies [23]. Moreover, a mixed meal tolerance test (MMTT)-based observational cohort study by Lazar et al has reported hypoglycemia (glucose level ≤54 mg/dL) prevalence as high as 88%, 82%, and 67% following RYGB, one-anastomosis gastric bypass (OAGB), and SG, respectively, with all surgeries performed more than one year before evaluation. Severe hypoglycemia (glucose level ≤40 mg/dL) was observed in 38%, 45%, and 7% of these groups, respectively. Despite these high rates of hypoglycemia identified by MMTT, only 24% of the postsurgery study participants experienced symptoms [42]. This finding suggests the possibility of either altered glycemic norms after surgery or the development of hypoglycemia unawareness in some individuals. In another cross-sectional study of randomly selected individuals who had undergone RYGB 4 years earlier, the prevalence of asymptomatic hypoglycemia (glucose level <3.3 mmol/L or 60 mg/dL) after an MMTT was 48% [43].

In a systematic review and meta-analysis by Lupoli et al, the weighted mean prevalence of PBH was 54.3%, with no significant difference between RYGB and SG. The prevalence of nocturnal hypoglycemia was 16.4%. Notably, studies evaluating PBH within the first year after surgery were excluded from this analysis [44]. Compared to previously reported prevalence rates of 0.1% to 34%, the recent findings suggest that PBH may be more prevalent than initially thought. Of particular concern are the high rates of hypoglycemia unawareness and nocturnal hypoglycemia, which may lead to underreporting and delayed diagnosis of PBH [42-45]. Data on PBH prevalence during pregnancy are limited. A retrospective observational study on 23 pregnant women who had undergone laparoscopic SG reported a PBH prevalence of 12.5% in those who became pregnant within 18 months of surgery and 6.7% in those who became pregnant after 18 months [46, 47]. A systematic review and meta-analysis by Stentebjerg et al found the PBH prevalence to be 57.6% in pregnant women after RYGB, as assessed by oral glucose tolerance test (OGTT) and CGM [48].

Several patient-related factors that can potentially increase the risk of PBH in nonpregnant individuals that have been reported in the literature are listed in Table 1 [49-54]. The specific risk factors associated with pregnancy are not yet known. Addressing this gap and identifying pregnancy-related risk factors could contribute to preventing PBH in pregnant women. The risk of developing PBH may also vary depending on the type of surgery and time since the procedure. Some studies suggest that the risk of PBH is greater with RYGB than with SG. In contrast, others report comparable prevalence rates between the 2 procedures but note higher glycemic variability with RYGB [44, 52, 55]. A more extended duration since the surgery can also increase the risk of PBH, highlighting the need for extended postoperative follow-up [44, 52].

**Table 1.** Risk factors for PBH reported in the literature

Younger age
Female gender
Higher insulin sensitivity, better β-cell glucose sensitivity
Lower preoperative HbA1c
Prior cholecystectomy
Absence of presurgery diabetes
Lower presurgery BMI
SSRI/SNRI use in RYGB patients without diabetes
Preoperative hypoglycemia symptoms/Low blood glucose at 120 minutes during postoperative OGTT
Younger women with significant weight loss after RYGB

Abbreviations: BMI, body mass index; HbA1c, glycated hemoglobin; OGTT, oral glucose tolerance test; PBH, post-bariatric hypoglycemia; RYGB, Roux-en-Y gastric bypass; SNRI, serotonin and norepinephrine reuptake inhibitors; SSRI, selective serotonin reuptake inhibitors.

## The Need for Evidence-Based Regional Consensus on the Diagnosis and Management of PBH

Our understanding of PBH, first described about 2 decades ago, as a post-bariatric surgery complication is gradually improving. With an increase in the number of MBS, especially with expanding eligibility criteria, the incidence of PBH is also likely to rise. Currently, there is significant heterogeneity in the definition, diagnosis, and treatment of PBH, leading to variability in clinical practice. The European Society for Endocrinology (ESE) recently published guidelines to standardize the diagnosis and management of PBH [23]. While these guidelines provide a comprehensive framework for PBH management, a GCC-specific perspective is essential to address the unique regional factors like extensive cultural diversity, dietary and lifestyle habits, access to specialist healthcare, diagnostic tools and treatments, and patient demographics. We believe that specialists practicing in the GCC region can utilize their collective experience with local patient populations, healthcare systems, and cultural factors to provide tailored guidance on PBH management in the region. To achieve these objectives, a task force comprising 16 obesity experts sought to (i) share their individual experiences and real-world insights on PBH management; (ii) identify challenges specific to PBH diagnosis and treatment in the GCC region; and (iii) develop consensus-based statements to address those challenges and guide PBH management in the GCC region.

## Methods

To ensure the representation of diverse perspectives, 16 experts from 7 countries were selected to form the task force panel. The panelists represented all the GCC countries (UAE, Saudi Arabia, Qatar, Bahrain, Kuwait, Oman) and an international obesity scientist. All panelists were carefully selected to represent the region's diverse expertise and included endocrinologists, obesity medicine physicians, bariatric surgeons, and a clinical nutritionist. Each panelist conducted a comprehensive literature search to identify relevant articles and ongoing trials related to PBH using PubMed, Google Scholar, and ClinicalTrials.gov (the National Institutes of Health clinical trial registry). The task force held discussions through virtual and in-person meetings to review the gathered evidence, collaboratively draft a set of statements on various aspects of PBH management and challenges, and organize them into relevant sections. The certainty of evidence for each statement was assessed using the GRADE methodology (Table S1) [56-58]. The statements, presented with their assigned certainty of evidence rating, were then evaluated through a Delphi survey using a 5-point Likert scale (1 = strongly disagree, 2 = disagree, 3 = neither agree nor disagree, 4 = agree, and 5 = strongly agree). Consensus was predefined as >80% of the panelists voting 4 and 5 on the Likert scale.

After the first round of voting, conducted on November 8, 2024, the task force met virtually on November 15, 2024, to discuss the voting results, further vet the statements, address the areas of disagreement, and refine them for the second round of voting. The statements were shared with the panelists on December 1, 2024, for a second voting round. The 2 rounds of voting were conducted using Google Forms. Voting results were collated and analyzed to ensure a consensus on all statements. Subsequently, the first draft of this manuscript was prepared to provide a rationale for the consensus statements and a clear, evidence-based, and clinical-experience-based framework for managing PBH. The panelists reviewed, discussed, and provided feedback via email to improve the draft. A collective input was actively sought from all panelists to reflect a unified GCC perspective toward PBH management. The draft underwent several iterations to ensure accuracy, clarity, and alignment with clinical evidence until all panelists approved it.

## Results

All 16 task force members participated in 2 rounds of voting on the expert statements through a Delphi survey. Voting was conducted anonymously, allowing panelists to express their opinions without influence. After the second round of voting, consensus was achieved on all 45 statements (see Tables 2-4, 6-8, and 10). Herein, we describe the rationale behind the expert statements organized into different sections, starting with “Screening for PBH” and ending with “Practical considerations for PBH management” to provide a logical flow to the PBH management pathway.

**Table 2.** Screening for PBH

Statement	Strongly agree	Agree	Neutral	Disagree	Strongly disagree	Consensus achieved?
Documenting detailed medical history (timing, frequency, and severity of hypoglycemia episodes, dietary triggers, medications, and surgical/medical history) is a crucial first step in patients suspected of PBH.	100%					Yes
PBH may be suspected in cases of postprandial symptomatic hypoglycemia that fulfill Whipple's triad criteria and usually present more than 1 year after bariatric surgery. Suspected cases should be evaluated to confirm the PBH diagnosis.	68.8%	38.3%				Yes
In cases of atypical PBH (hypoglycemia occurring less than 1 year post-bariatric surgery and/or during fasting), other possible causes of hypoglycemia should be excluded.	50%	37.5%	12.5%			Yes

Abbreviation: PBH, post-bariatric hypoglycemia.

**Table 3.** Diagnostic confirmation of PBH

Statement	Strongly agree	Agree	Neutral	Disagree	Strongly disagree	Consensus achieved?
The diagnosis of PBH is confirmed if the following criteria are met: Hypoglycemia (blood glucose < 54 mg/dL [3 mmol/L]) occurring within 1-3 hours after meals and fulfilling Whipple's triad History of bariatric surgery more than 1 year prior to hypoglycemia episodes Absence of fasting hypoglycemia (defined as > 8 hours of non-caloric food intake) Medication history confirming no recent use of hypoglycemia-inducing agents	68.8%	25%	6.3%			Yes
Patients not fulfilling the criteria specified in statement #1 should be referred for specialist care at Bariatric, Obesity, or Endocrinology centers with experience in managing PBH.	100%					Yes
Since venous plasma glucose measurement may not be feasible every time hypoglycemia is suspected, capillary blood glucose (CBG) may be considered a reliable and practical alternative for the diagnosis of PBH.	50%	43.8%	6.3%			Yes
A regulatory agency-approved continuous glucose monitoring (CGM) system may be considered in selected symptomatic patients where capillary blood glucose (CBG) use is limited due to patient-related factors. However, a countercheck with CBG at the time of hypoglycemia is required to confirm the glucose level.	62.5%	31.3%	6.3%			Yes
Continuous glucose monitoring (CGM) may also be considered to identify patients experiencing “nocturnal hypoglycemia.”	68.8%	31.3%				Yes
A mixed meal tolerance test (MMTT) is not recommended to diagnose PBH.	43.8%	37.5%	6.3%	6.3%	6.3%	Yes
The oral glucose tolerance test (OGTT) is not recommended for the diagnosis of PBH.	75%	12.5%	6.3%		6.3%	Yes
Assessment of cortisol axis deficiency should be considered whenever clinically suspected.	68.8%	25%		6.3%		Yes

Abbreviation: PBH, post-bariatric hypoglycemia.

**Table 4.** Dietary, pharmacological, and surgical management of PBH

Statement	Strongly agree	Agree	Neutral	Disagree	Strongly disagree	Consensus achieved?
***Dietary management of PBH***						
Proactive management of PBH with individualized treatment plans and a readiness to adjust strategies as needed without clinical inertia is essential.	87.5%	12.5%				Yes
Dietary modification should always be considered as a first-line therapy before pharmacotherapy in the management of PBH.	87.5%	12.5%				Yes
Dietary modifications should be made under the supervision of a registered dietitian with experience in PBH management who can personalize the nutritional plans and monitor other additional nutritional deficiencies.	93.8%	6.3%				Yes
***Importance of patient education in dietary management of PBH***						
Dietary modification education should be a continuum of focused training sessions over 1-2 weeks after diagnosis, with frequent re-enforcement sessions, especially in the first 3-6 months.	75%	18.8%	6.3%			Yes
Pharmacotherapy options should be considered if dietary changes are ineffective over a period of 12 weeks.	62.5%	37.5%				Yes
An earlier combined dietary/pharmacological intervention may be considered for severe, intractable symptoms.	75%	25%				Yes
***Special considerations during Ramadan***						
During Ramadan, patients must be counseled to adjust the composition and quantity of their Iftar and Suhoor meals. They should be encouraged to minimize and space out meals, avoid high-carb or sugar content, chew food properly, and minimize/avoid juice at Iftar. The management of PBH should be guided by general dietary principles for post-MBS individuals as well as specific nutritional guidance for PBH.	57.1%	28.6%	7.1%		7.1%	Yes
**Pharmacological and surgical management of PBH** ***Pharmacological treatment of PBH***						
Acarbose, an α-glucosidase inhibitor, in 2-3 divided doses per day, should be considered a first-line pharmacological therapy for treating PBH unless contraindicated.	43.8%	50%	6.3%			Yes
Appropriate education on the potential side effects of acarbose may improve adherence and tolerance to its adverse effects.	37.5%	50%	12.5%			Yes
Short or long-acting pasireotide or short-acting SC octreotide may be considered a second-line option if acarbose is ineffective in treating PBH over a period of 12 weeks.	43.8%	37.5%	18.8%			Yes
Based on the current evidence, the lowest effective dose of somatostatin analogues is preferred.	43.8%	43.8%	12.5%			Yes
Due to the absence of head-to-head randomized studies comparing pasireotide and octreotide, the decision regarding the route of administration and choice of therapy should be dictated by drug availability and physician preference.	31.3%	62.5%	6.3%			Yes
Diazoxide, a potassium channel activator, may be considered as a third-line therapy after assessing its risk-benefit profile.	43.8%	37.5%	18.8%			Yes
GLP-1 receptor agonists may be considered in treating PBH if other available pharmacotherapy options are ineffective or unavailable, and especially if further weight loss is desired.	31.3%	50%	12.5%	6.3%		Yes
Glucagon can be considered to treat acute hypoglycemia episodes in certain patients with severe and frequent PBH.	56.3%	31.3%	12.5%			Yes
***Surgical management of PBH: a last resort option***						
Surgical interventions should be considered after thoroughly evaluating their risks and benefits on a case-by-case basis.	62.5%	25%	12.5%			Yes
Surgical interventions, such as laparoscopic/endoscopic reversal of bariatric surgery, should be considered as the last resort in medically refractory cases or severe PBH with hypoglycemia unawareness.	43.8%	43.8%	6.3%	6.3%		Yes

Abbreviations: GLP-1, glucagon-like peptide 1; PBH, post-bariatric hypoglycemia; SC, subcutaneous.

**Table 5.** Dietary modification for PBH

Recommended diet plan: Small and frequent (every 3-4 hours) meals are recommended. Meals should comprise all 3 macronutrients (fats, proteins, and carbohydrates). Low-glycemic carbohydrates (<30 g/meal and <15 g/snack) Adequate amount of protein intake: 1.5-2.1 g/kg of ideal body weight depending on the activity level (ie, ranging from 1.5 g/kg for those who are not very physically active to 2.1 g/kg for physically active adults) Healthy fats in each meal (15 g/meal and 5 g/snack) Adequate dietary soluble fiber should be consumed with each meal. Liquids should not be consumed during meals and should be consumed at least 30 minutes after meals. Alcohol should be avoided. The effect of caffeine may be evaluated on a case-by-case basis, and recommendations tailored accordingly.
The diet plan is based on limited evidence underscoring the importance of closely monitoring PBH patients after initiating dietary modifications and making necessary adjustments to achieve optimal outcomes.
The dietary plan should be personalized based on patient-related and clinical factors in collaboration with a registered dietician.
The dietary plan should achieve an optimal balance between addressing potential post-bariatric surgery nutritional deficiencies and managing PBH.
PBH patients’ behavioral and psychological aspects should be considered when deciding on their dietary plans.
Patients should be counseled regarding the possible need to adjust their dietary plans depending on their glycemic and PBH symptom patterns.

**Table 6.** Considerations during pregnancy

Statement	Strongly agree	Agree	Neutral	Disagree	Strongly disagree	Consensus achieved?
All pregnancies after metabolic and bariatric surgery are considered high-risk, including those complicated by PBH.	81.3%	18.8%				Yes
Management of PBH during pregnancy may be very challenging; we advise referral to an endocrinologist with vast experience in the management of complicated PBH.	87.5%	12.5%				Yes
PBH may impact fetal growth, increasing the risk of small-for-gestational-age (SGA) births, thus necessitating careful monitoring and management during pregnancy to ensure improved fetal outcomes.	62.5%	31.3%	6.3%			Yes
Dietary modification remains the first tool in the management of PBH in pregnancy.	81.3%	18.8%				Yes
Based on a thorough assessment of its risk-benefit profile, acarbose may be considered in the treatment of PBH during pregnancy after securing the patient's agreement and informed consent to initiate or continue its administration.	56.3%	37.5%		6.3%		Yes
Oral glucose tolerance test (OGTT) is used routinely to detect hyperglycemia during pregnancy. However, in cases of PBH in pregnancy, the risk of hypoglycemia post-glucose load is high. Therefore, it is recommended to avoid it during pregnancy with PBH.	50%	43.8%		6.3%		Yes

Abbreviation: PBH, post-bariatric hypoglycemia.

**Table 7.** Long-term management and monitoring

Statement	Strongly agree	Agree	Neutral	Disagree	Strongly disagree	Consensus achieved?
Close monitoring of patients after treatment initiation is necessary to evaluate outcomes and check for potential side effects.	81.3%	18.8%				Yes
a. A regulatory agency-approved continuous glucose monitoring (CGM) may be used to assess the effectiveness of dietary and pharmacotherapy interventions in the management of PBH. b. The use of a regulatory agency-approved CGM system may be considered to monitor patients who may be experiencing hypoglycemia unawareness.	**81.3%** **87.5%**	**18.8%** **6.3%**	6.3%			**Yes** **Yes**
As PBH can adversely impact patients’ overall quality of life and psychological well-being, involvement of a psychologist with experience in obesity medicine is warranted whenever clinically indicated.	68.8%	18.8%	12.5%			Yes
Technology, mobile applications, and remote education/teleconsultation might play a role in maintaining long-term follow-up. They could be utilized more in monitoring PBH after intervention.	50%	37.5%	12.5%			Yes

Abbreviation: PBH, post-bariatric hypoglycemia.

**Table 8.** Unmet needs and emerging approaches in PBH management

Statement	Strongly agree	Agree	Neutral	Disagree	Strongly disagree	Consensus achieved?
More research is needed to understand the glycemic variability throughout the day in PBH patients.	56.3%	43.8%				Yes
There is a lack of head-to-head comparative studies between pharmacotherapies currently used for PBH, underscoring the need to conduct robust clinical trials to address this gap.	81.3%	18.8%				Yes
More effective pharmacotherapeutic options for PBH treatment are needed.	68.8%	31.3%				Yes
Continuous glucose monitoring using novel tools holds promising potential for improving PBH diagnosis and monitoring treatment outcomes when combined with a detailed food and symptoms diary.	56.3%	37.5%	6.3%			Yes

Abbreviation: PBH, post-bariatric hypoglycemia.

**Table 9.** Emerging therapies currently under investigation

Drug/System	Type of drug/system	Type of study	Main finding
Tirzepatide	Dual GLP-1-GIP receptor agonist/ Dual incretin agonist	Case report No ongoing clinical trial	Efficacious option, especially for patients with PBH after SG.
Avexitide [exendin (9-39)]	GLP-1 receptor antagonist	Multicenter, phase 2, randomized, placebo-controlled crossover study (PREVENT)	Compared to placebo, avexitide 30 and 60 mg raised glucose nadir by 21% and 26% and reduced insulin peak by 23% and 21%. CGM showed reduction in hypoglycemia without triggering clinically relevant hyperglycemia.
Mizagliflozin	SGLT1 inhibitor	Randomized, sequential crossover single dose study	Mean glucose nadir change from baseline was 12.6 ± 22.5 mg/dL
Canakinumab	IL-1 receptor antagonist	National (Switzerland), multicenter, phase 3, randomized, placebo-controlled, parallel-group, double-blind superiority trial	Ongoing study
CGM-guided closed loop glucagon system	Glucose-responsive glucagon delivery system	Randomized, placebo-controlled, masked phase 1 and 2 trial	The CGM-guided system could predict a hypoglycemia event based on declining glucose levels and deliver a rescue dose of glucagon.
Dasiglucagon	Stable glucagon analogue in a ready-to-use formulation	Randomized, double-blind, placebo-controlled, crossover, proof-of-concept study	Compared to placebo, 4 weeks of self-administered dasiglucagon reduced clinically relevant hypoglycemia post-RYGB surgery.

Abbreviations: CGM, continuous glucose monitoring; GLP-1, glucagon-like peptide 1; GIP, gastric inhibitory peptide; PBH, post-bariatric hypoglycemia; RYGB, Roux-en-Y gastric bypass; SG, sleeve gastrectomy; SGLT1, sodium-glucose transporter 1.

**Table 10.** Practical considerations in PBH management

Statement	Strongly agree	Agree	Neutral	Disagree	Strongly disagree	Consensus achieved?
Patients should routinely be informed about potential postoperative challenges and nutritional requirements, including PBH, and evaluated for their readiness to adopt behavioral modifications.	81.3%	18.8%				Yes
Secondary or tertiary care institutions should be encouraged to recruit/train nutritionists to appropriately manage patients with PBH.	68.8%	31.3%				Yes
Additional regulations and licensing requirements should be implemented for issuing driving licenses or employing patients with PBH in high-risk jobs (eg, operating heavy machinery) if they lack access to continuous glucose monitoring due to the potential risk of hypoglycemia unawareness.	50%	43.8%	6.3%			Yes

Abbreviation: PBH, post-bariatric hypoglycemia.

### Screening for PBH

A thorough medical history is vital for accurate diagnosis. Documenting the nature, timing, and frequency of hypoglycemia symptoms—the presence of neuroglycopenia, whether the symptoms occur during fasting or after meals, and how often they occur—can help identify specific trends in hypoglycemic episodes [59]. Encouraging individuals who are undergoing bariatric surgery to maintain a food and symptom diary can help identify dietary triggers contributing to symptoms [23]. These are easy-to-implement practices for physicians and patients that can help in symptom tracking and contribute to the first step in accurately diagnosing PBH. Moreover, it is necessary to confirm that patients fulfill Whipple's triad criteria (hypoglycemia symptoms, plasma glucose levels <54 mg/dL, and symptom relief after glucose administration) to ensure that their symptoms are caused by hypoglycemia [60].

Due to numerous confounding factors such as rapid weight loss and evolving metabolic changes in the immediate postoperative period, most retrospective and prospective studies have evaluated PBH incidence once patients are metabolically stable ≥1 year after MBS [44, 54, 61-65]. Based on our experience also, PBH typically presents more than 1 year after MBS. Thus, patients presenting with postprandial hypoglycemia symptoms more than 1 year after an MBS, with hypoglycemia confirmed by Whipple's triad criteria, should be evaluated further to confirm the diagnosis. By recognizing the clinical signs of PBH early, physicians can initiate the necessary steps to confirm or rule out the diagnosis and provide timely intervention. Additionally, in patients presenting with hypoglycemia symptoms within 1 year of MBS, ruling out other possible causes of hypoglycemia (eg, insulinoma, nutritional or hormonal deficiencies, medications, extra-islet tumors, nesidioblastosis, etc.) is essential to avoid misdiagnosis or missed diagnoses [23, 66, 67]. Improving physician awareness of PBH is necessary to enable them to recognize the clinical symptoms and signs.

### Diagnostic Confirmation of PBH

In suspected cases of PBH, diagnosis should be confirmed through a combination of clinical presentation and laboratory findings. Typical PBH presentation includes postprandial hypoglycemia that occurs 1 to 4 hours after meals, along with symptoms associated with sympathetic nervous system activation and neuroglycopenia (see Fig. 1) [23, 45, 59]. Based on this classical PBH presentation, diagnosis can be confirmed if patients meet these 4 criteria: (i) symptomatic postprandial hypoglycemia that fulfills Whipple's triad; (ii) a history of MBS performed more than 1 year before symptom onset; (iii) absence of fasting hypoglycemia to rule out other causes; and (iv) no recent use of hypoglycemia-inducing drugs.

While it may be relatively straightforward to identify typical PBH cases, physicians should also be vigilant in recognizing and addressing unusual presentations. They should immediately refer patients not meeting the primary diagnostic criteria to specialist centers with expertise in managing PBH. Physicians should be aware that although PBH typically manifests more than 1 year after MBS, it can present earlier in some cases [68, 69]. Also, although PBH usually occurs postprandially, cases of hypoglycemia during fasting have been reported [70, 71]. Interestingly, an analysis by Lupoli et al found that postprandial, symptomatic hypoglycemia is more common after RYGB, whereas hypoglycemic episodes tend to be asymptomatic and nocturnal after SG [44, 72]. Further research on this aspect would help to stratify patients based on their PBH risk and facilitate diagnosis and personalized treatment. Referral of complex and atypical cases, where PBH symptoms may overlap with other disorders, to specialist centers can prevent delays in diagnosis and reduce the risk of misdiagnoses.

PBH diagnosis relies on accurately measuring blood glucose levels during hypoglycemic episodes. Nonetheless, while choosing a diagnostic approach, it is also crucial to balance the accuracy of standardized laboratory venous blood glucose measurement with the practicality and acceptable reliability of capillary blood glucose (CBG) measurement done by a qualified healthcare provider [73, 74]. Recommendations published previously by the ASMBS in 2017 and the Obesity Group of the Spanish Society of Endocrinology and Nutrition (GOSEEN) in 2021 have emphasized using venous blood glucose measurements to assess glycemic levels accurately [45, 75]. However, the guidelines published by the Society for Endocrinology in 2024 recommend a more pragmatic approach [23]. CBG measurements may be less precise, but they are accepted in clinical practice due to their ease of use and accessibility when venous blood glucose assessment is not easily accessible. We agree with this practical approach, which does not significantly compromise diagnostic accuracy and is better suited for the GCC region. We also note that the accuracy of glucometers is influenced by variable user techniques (eg, size of the blood drop, testing site), environmental conditions (eg, heat, moisture), and the presence of other substances in the blood (eg, uric acid, triglycerides, medications) [76]. Apart from these factors, multiple measurements confirming glucose levels <54 mg/dL [3 mmol/L], using a validated (ie, meeting ISO 15197:2013, FDA, or other equivalent regulatory agency standards) CBG device, must be considered before confirming a PBH diagnosis [77].

The development of CGM using implantable sensors has allowed real-time continuous or intermittent (flash glucose monitoring), depending on the device, tracking of glycemic excursions throughout the day, including postprandial and nocturnal periods. A multicenter prospective study evaluating the CGM profiles of healthy nondiabetic participants (ages 7 to 80) found that hypoglycemic episodes (glucose levels <54 mg/dL [3.0 mmol/L]) were uncommon in this cohort. Moreover, glucose levels <70 mg/dL were encountered infrequently during the nighttime [78]. These daily glycemic trends monitored using CGM technology have established a normal baseline reference consistent with what would be expected in a healthy population. However, the accuracy of CGM systems in the hypoglycemic range has been questioned in a systematic review and meta-analysis by Lindner et al, suggesting that they may not be a reliable substitute for more established diagnostic methods like CBG [79]. Thus, the use of CGM in the diagnosis of PBH remains debatable, which is why the recent Society for Endocrinology guidelines do not recommend it for diagnosing PBH but suggest it may only be used to improve PBH management.

While we agree that CGM should not exclusively be used to diagnose PBH, we support its use as an adjunct to CBG measurements in select cases for the following 2 reasons. First, CGM allows us to monitor the duration of hypoglycemic episodes over an extended period, providing a comprehensive overview of glycemic trends. This capability offers a better snapshot of glucose fluctuations related to daily activities, including meals, exercise, and sleep, compared to point-of-care methods like CBG [76, 80, 81]. Second, nocturnal PBH may be more prevalent than initially thought, underscoring the need to ensure it is not overlooked during diagnosis [44, 72]. Thus, it is reasonable to consider CGM as a tool to identify potential post-bariatric nocturnal hypoglycemic episodes. It may also be used in select symptomatic patients for whom multiple CBG measurements by a qualified healthcare provider may not be feasible. However, final confirmation of PBH diagnosis should rely on CBG confirmation at the time of hypoglycemia.

OGTT and MMTT are provocation tests that use different stimuli to assess glucose metabolism. OGTT evaluates blood glucose levels at baseline and then at different time points after an administration of 75 g of glucose [82]. It is routinely used in the diagnosis of diabetes. However, it is not recommended for diagnosing PBH because it: (i) involves consuming a glucose solution, which does not replicate typical meal components, to evaluate response; (ii) is associated with a high incidence of side effects in post-bariatric individuals; and (iii) shows a poor association between reported hypoglycemia symptoms and low blood glucose levels detected during the test [16, 23, 31, 75, 83, 84].

As opposed to a single-component-based OGTT, MMTT uses a liquid or solid meal comprising fats, carbohydrates, and proteins. Significant variability exists in the published literature regarding the utility of MMTT in PBH diagnosis. In contrast with the previous recommendations by the ASMBS and GOSEEN, the latest Society for Endocrinology guidelines do not recommend MMTT [16, 23, 75]. Given the heterogeneity in test protocols, including meal composition and patient factors (eg, time from surgery) that can influence the diagnostic outcomes, we agree with the Society for Endocrinology recommendation [23, 85]. Although it has been suggested that MMTT provides a more physiologically similar stimulus compared to the OGTT, it still differs from the real-world dietary patterns and conditions that typically trigger hypoglycemia in post-bariatric individuals. Thus, we do not recommend using MMTT in PBH diagnosis. Despite its limitations, we acknowledge that MMTT continues to be used in some cases. In these cases, a negative MMTT should not be used to rule out PBH and should prompt additional investigations.

Cortisol and other counterregulatory hormonal responses to hypoglycemia are diminished in post-bariatric individuals [86-89]. Furthermore, symptoms of PBH overlap with those of adrenal insufficiency, complicating the diagnosis. Although rare, cases of post-bariatric hypocortisolism (adrenal insufficiency) after biliopancreatic diversion, RYGB, and SG have been reported in the literature [90-93]. Thus, if clinically suspected, assessment of cortisol axis deficiency should be considered. If left undetected, cortisol axis deficiency can exacerbate hypoglycemia and may have serious consequences.

### Management of PBH

**Figure 3.**
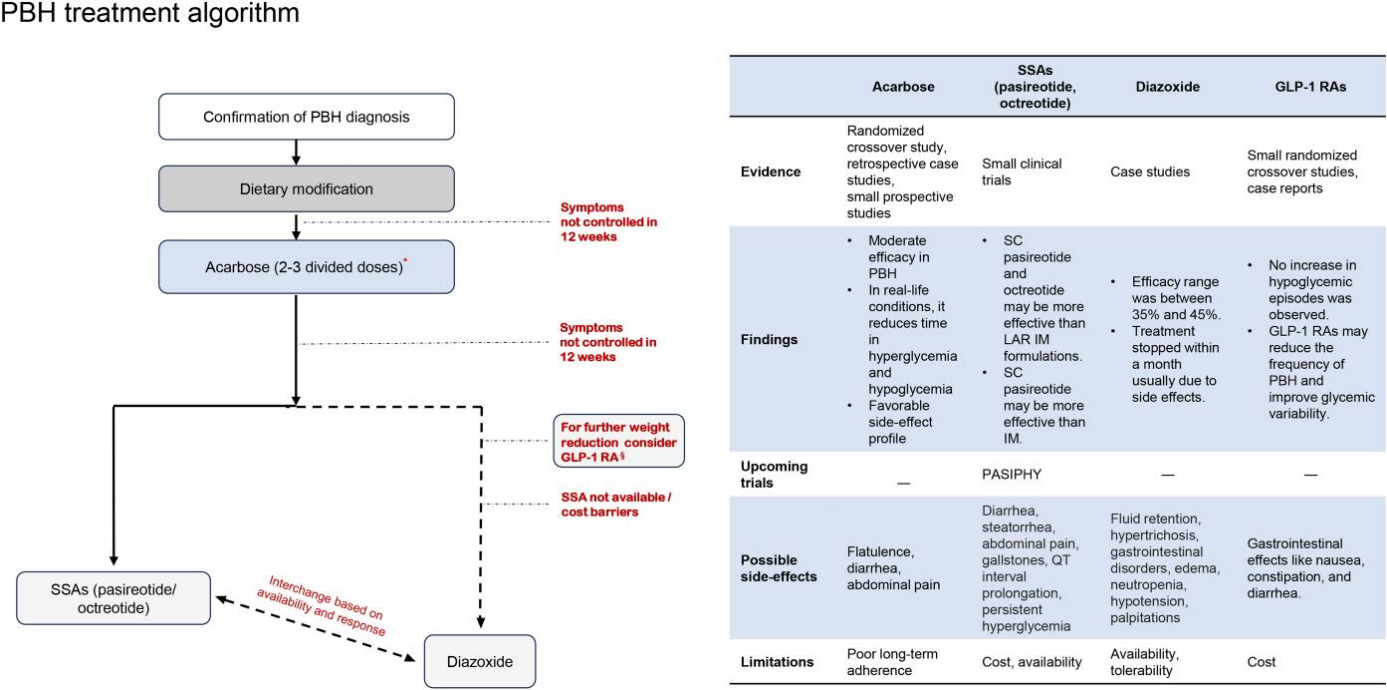
PBH treatment algorithm. *Could be initiated earlier in case of severe intractable symptoms. ^§^There is limited evidence on the use of GLP-1 RAs; however, experts agreed on the potential clinical benefit, especially when weight remains a concern, and SSAs are unavailable. Abbreviations: GLP-1 RA, glucagon-like peptide 1 receptor agonist; IM, intramuscular; LAR, long-acting release; PBH, post-bariatric hypoglycemia; SC, subcutaneous; SSAs, somatostatin analogues.

Timely diagnosis and effective management of PBH require a proactive approach tailored to address patients’ specific needs. Physicians should regularly follow up with PBH patients, monitor their progress, and modify treatments promptly when necessary. This proactive approach in PBH management is crucial in preventing future complications and improving outcomes (see Fig. 3 and Table 4). A consolidated visual management algorithm is provided in Fig. S1 [58].

#### Medical Nutrition Therapy

Medical nutrition therapy is an integral part of interventions targeting metabolic disorders. Thus, we agree with all the previous consensus statements recommending dietary modification as the first step in treating PBH [23, 45, 75]. It is a noninvasive intervention that may be more sustainable in the long term in compliant patients. Dietary modification should primarily focus on reducing the postprandial glucose spike that triggers the pathological cascade leading to hypoglycemia [94]. The dietary modifications recommended by the expert panel are detailed in Table 5 [23, 28, 95, 96]. The proposed nutritional plan is based on evidence from small, uncontrolled, real-world, and retrospective studies, and our clinical experience. Optimal protein, fat, and carbohydrate intake in each meal is crucial to balance meeting nutritional and energy needs and preventing hypoglycemia. Studies have shown that restricting carbohydrates to <30 g/meal can reduce hypoglycemia symptoms [23, 97, 98]. Although robust studies comparing the effect of low vs high glycemic index meals in the context of PBH are lacking, low glycemic index carbohydrates are recommended, as they do not cause rapid glucose spikes and are therefore less likely to trigger hypoglycemia in PBH patients [28]. Protein intake should be tailored based on activity level, ranging from 1.5 g/kg of ideal body weight for those with low physical activity to 2.1 g/kg of ideal body weight for physically active adults [28, 95]. Including moderate amounts (15 g/meal and 5 g/snack) of heart-healthy fats is recommended, as fats do not independently trigger insulin secretion and can provide sustained energy [28, 95]. Additionally, consuming small, frequent meals and avoiding liquids during meals is recommended to prevent rapid gastric emptying in PBH patients. This recommendation has been reinforced by evidence from a prospective study by Stano et al, which evaluated the effect of meal texture and size on gastric pouch emptying and GLP-1, insulin, and glucose levels before and after RYGB. After RYGB, hypoglycemia was observed more frequently with large meals and liquid meals compared to small and/or solid meals [99]. Although the effect of caffeine on PBH has not been studied, it can lead to increased blood glucose levels in sensitive individuals [28, 95]. Thus, evaluating its effect on a case-by-case basis may be advisable, with recommendations tailored accordingly. As alcohol reduces hepatic gluconeogenesis, PBH patients should be recommended to avoid alcohol to prevent the risk of hypoglycemia [23, 95].

While the recommendations provided here are broadly applicable to all PBH patients, they must be tailored to meet individual nutritional needs and address specific deficiencies. Patient factors, such as weight and level of physical activity; clinical factors including vitamin and mineral deficiencies or eating disorders (eg, binge eating, bulimia, etc.); and cultural factors such as traditional dietary practices should be considered when tailoring diet plans for PBH patients. Collaborating with a registered dietician experienced in managing PBH can help achieve this.

Providing patients with a diet plan, no matter how detailed, will not be effective if they do not comply with it. Instructing patients to modify their eating habits without a clear understanding of why it is necessary can lead to poor adherence. Studies in the Gulf region highlight a significant impact of cultural dietary habits on the local population. A large number of individuals diagnosed with diabetes in this region continue to overlook food labels and struggle to differentiate between low and high glycemic index foods, implying a gap in diet-related education [100]. These challenges are also likely to be relevant for patients with PBH.

#### Importance of Patient Education in Dietary Management of PBH

A reluctance to modify diet and behavior toward food habits could be a significant barrier to improving outcomes through dietary interventions in PBH patients. Several studies show the significant positive impact of nutrition education programs on outcomes in several chronic conditions, including metabolic syndrome [101-107]. Svetkey et al showed that maintaining positive outcomes, such as weight loss achieved through a focused behavioral weight loss intervention, can be difficult. However, personal counseling and support with a frequent reinforcement strategy helped participants in this study better maintain weight loss compared to a self-directed control group [108].

**Figure 4.**
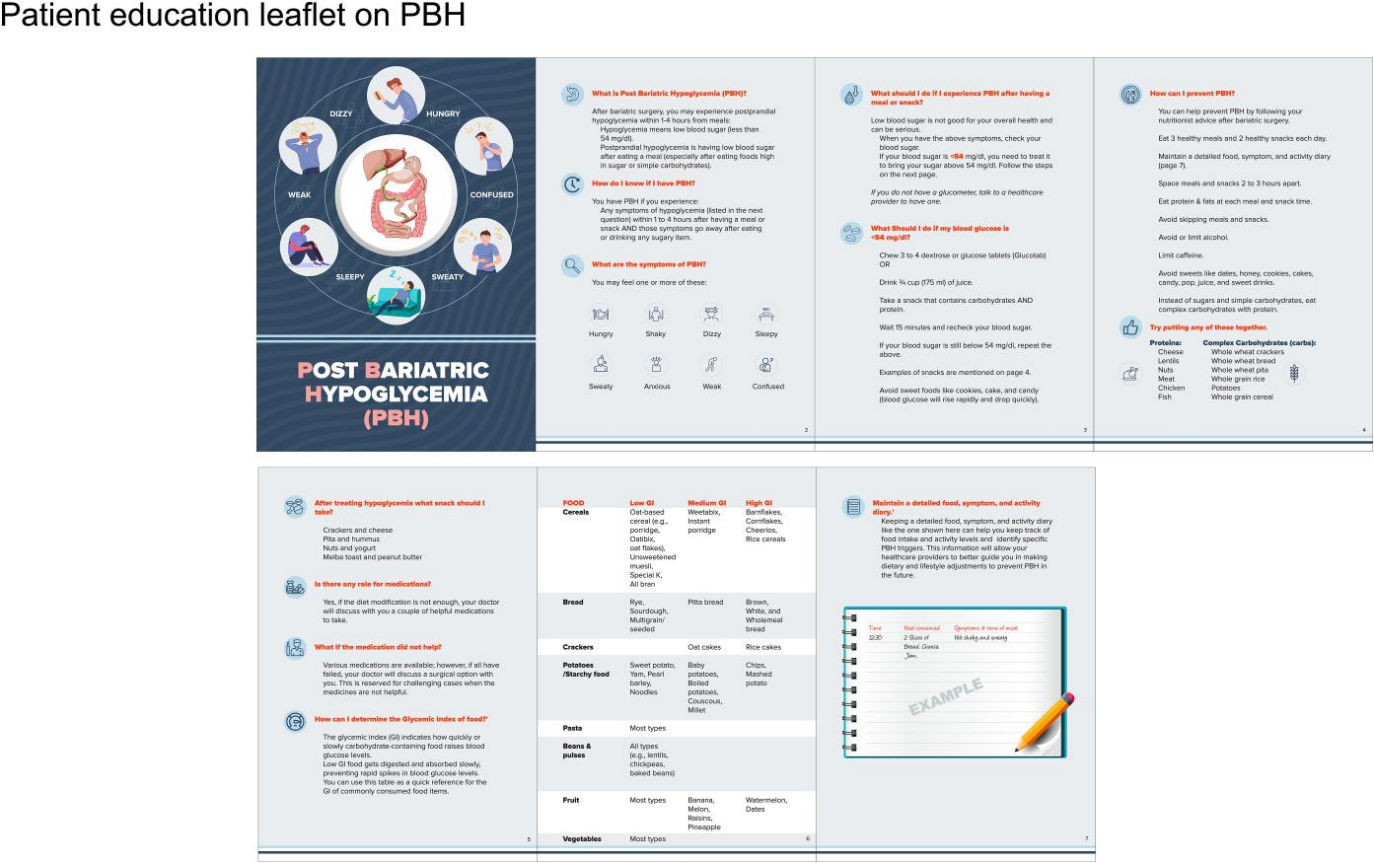
Patient education leaflet on PBH.

We recommend a structured program involving informative sessions that emphasize adherence to the prescribed diet plan. These sessions should also highlight the clinical implications of not complying with the diet plan. The training sessions can be conducted over 1 to 2 weeks after PBH diagnosis, followed by frequent reinforcement sessions, especially during the first 3 to 6 months, to sustain motivation and ensure adherence to dietary recommendations. Additionally, patients may be provided with an informational brochure (education leaflet, also translated into local Arabic/Urdu languages) (see Fig. 4) on managing PBH, including symptoms of hypoglycemia to watch for, blood glucose assessment, dietary suggestions to prevent hypoglycemia, and acute treatment strategies during a hypoglycemic episode.

Dietary modification alone is often insufficient for managing moderate to severe cases of PBH [109]. Typical clinical practice suggests that a 3-month (12-week) trial would be optimal to assess patients’ adherence and response to dietary changes. If symptoms persist after 12 weeks of consistent adherence to the recommended diet plan, pharmacotherapy should be considered to ensure early intervention. Moreover, the 12-week time frame is just a practical guideline, and an earlier pharmacological intervention may be warranted in PBH patients presenting with severe, intractable symptoms.

#### Special Considerations During Ramadan

In the absence of studies defining optimal pre-fast and post-fast meal composition during prolonged fasting, such as during Ramadan, management of PBH should be guided by general dietary principles for post-MBS individuals as well as specific nutritional guidance for PBH (see Table 5). Consuming a low glycemic index, high-fiber, high-protein diet before the fasting period can allow slow, sustained glucose release and ensure optimal energy levels throughout the day. Similarly, when breaking the fast, PBH patients should be counseled to avoid overeating and consuming food rapidly, to ensure food is chewed properly, and to divide the Iftar meal into 2 to 3 smaller portions consumed over several hours [95, 110, 111].

#### Pharmacological Treatment of PBH

There is currently no approved pharmacological therapy for PBH. However, some medications have been evaluated in small clinical trials and case studies or have limited real-world evidence supporting their use in PBH management. Figure 3 shows a treatment algorithm for these medications [23, 112-115].

##### Acarbose

Acarbose is a competitive and reversible inhibitor of the intestinal α-glucosidase, which breaks down carbohydrates into monosaccharides. In a randomized crossover study on 11 PBH patients by Ohrstrom et al, 50 mg acarbose administered 4 to 6 times a day for 1 week reduced the postprandial hyperglycemic peak and improved hypoglycemia [116]. Analysis of retrospective data on 120 patients with a median follow-up of 27 months showed a 100% reduction of hypoglycemia in 21% of patients and 50% to 100% resolution in 35% of patients [114]. Based on this and other previously published evidence, acarbose can be considered a first-line pharmacological treatment for PBH in patients for whom dietary modifications alone prove insufficient (see Fig. 3) [23, 114, 116-119]. However, gastrointestinal side effects of acarbose can lead to treatment discontinuation [109]. Hence, informing patients about the potential side effects of acarbose before initiating treatment may help set realistic expectations and improve adherence.

##### Somatostatin analogues

Somatostatin analogues (SSAs) are synthetic versions of somatostatin (SST), a cyclic polypeptide that inhibits several endocrine and exocrine hormones, including those involved in glucose homeostasis, like insulin and glucagon [120]. Although the FDA has not yet approved SSAs for treating PBH, data from studies on the first-generation SSA, octreotide, and second-generation SSA, pasireotide, suggest their potential efficacy in managing PBH [23, 28, 120, 121]. Both octreotide and pasireotide are available in subcutaneous (SC) and long-acting intramuscular (IM) formulations.

In an open-label study, 29 patients with postoperative dumping, who were nonresponsive to dietary interventions, were treated with SC octreotide for 3 days, followed by monthly IM octreotide long-acting repeatable (LAR) formulation for 3 months. Both formulations significantly improved late dumping symptoms and QoL. However, the overall severity score for late dumping symptoms was significantly lower in patients treated with SC octreotide than those treated with octreotide LAR. Also, in patients with an intact gallbladder, the increased risk of gallstones associated with octreotide LAR offsets the convenience of its once-monthly dosing [122]. Long-term follow-up study of patients treated with SC or LAR octreotide showed that nearly half of the patients discontinued treatment due to either loss of efficacy or side effects. The findings also suggested that despite the convenience of once-monthly administered IM formulation, the daily-administered SC formulation may offer a more favorable risk-benefit profile [123].

Pasireotide, another SSA, exhibits greater inhibition of insulin secretion and incretin effect and a slight reduction in glucagon levels [124]. These actions likely contribute to pasireotide's more pronounced hyperglycemic effects [125]. A single-arm, open-label, multicenter study on 38 post-bariatric participants with late dumping (PBH) evaluated the efficacy of pasireotide in reducing hypoglycemic episodes. The patients were treated with SC pasireotide for 3 months, followed by IM pasireotide for 3 months, with an option to extend the IM phase treatment to 6 months. The study findings revealed that SC pasireotide 50 µg to 200 µg could prevent postprandial hypoglycemia by lowering glucose-dependent insulinotropic polypeptide (GIP), GLP-1, and insulin levels. Moreover, patient QoL parameters that had improved during the SC phase of the trial were maintained in the IM phase, and no unexpected safety signals were identified. Hyperglycemia as an adverse effect was relatively less common in this cohort than in other patient populations [121]. An evaluation of the acute effects of pasireotide at doses of 75 µg, 150 µg, and 300 µg revealed that all doses reduced insulin, C-peptide, and GLP-1 responses [126]. Based on these data, the lowest effective dose of SC or LAR pasireotide, or SC octreotide, may be considered in patients who show no response to acarbose after 12 weeks (see Fig. 3).

Although a head-to-head comparison of pasireotide LAR and octreotide LAR in medically naïve acromegaly patients has demonstrated superior efficacy of pasireotide LAR, similar studies have not been conducted in the PBH patient population [125]. In the absence of such studies, the choice of treatment and route of administration should be guided by drug availability, cost, and physician preference.

##### Diazoxide

Diazoxide binds to the sulfonylurea receptor-1 subunit of the ATP-sensitive potassium channel and inhibits insulin secretion [127]. Studies on the successful use of diazoxide in PBH are limited to case reports and case series, and clinical trial evidence on its efficacy is lacking [114, 128-130]. Despite the small body of evidence, diazoxide has been used for individuals with severe, intractable PBH by specialist centers, including those in the GCC region. Some case reports have also demonstrated its effective use in postprandial hypoglycemia after primary gastric surgery [131]. However, the use of diazoxide may be limited by adverse effects such as fluid retention, hypertrichosis, gastrointestinal disorders, hypotension, edema, and neutropenia, which warrant careful monitoring [28, 112]. Given the availability of only limited pharmacotherapy options in PBH, diazoxide may be considered third-line in patients with persistent and severe symptoms who do not respond to acarbose and SSAs. It may also be considered following acarbose treatment if symptoms remain persistent, uncontrolled, and severe, and SSAs are either unavailable or pose cost barriers (see Fig. 3).

##### Glucagon-like peptide 1 receptor agonists

GLP-1, an incretin hormone secreted by intestinal L cells, delays gastric emptying, and stimulates insulin secretion from beta cells in response to food intake. The insulinotropic effects of GLP-1 depend on blood glucose levels and GLP-1 concentration. Bariatric surgery causes rapid food transit, leading to an early and significant increase in GLP-1 levels. The GLP-1 levels are elevated after bariatric surgery and are higher in PBH patients compared to those who do not develop hypoglycemia [115]. A systematic review of 6 studies published between 2013 and 2021 conducted by Llewellyn et al showed that there is limited evidence to suggest that GLP-1 receptor agonists (GLP-1 RAs) may help reduce the number of postprandial hypoglycemic episodes and improve glycemic variability in patients experiencing hyperinsulinemic hypoglycemia syndrome following bariatric surgery [115]. Although the precise role of GLP-1 RAs in preventing hypoglycemic episodes remains unclear, and it would be premature to draw conclusions, a recently published case report and a small retrospective medical chart review study also suggest a potential role for GLP-1 RAs in PBH [132, 133]. Thus, given the role of GLP-1 RAs in inducing weight loss, they may be considered when other pharmacotherapy options are ineffective or unavailable, especially if further weight loss is desired or in case of suboptimal weight reduction after surgery [115]. The decision should be based on individualized assessment of potential risks vs benefits. Physicians should exercise caution and initiate treatment within specialist centers to ensure oversight. (see Fig. 3).

##### Glucagon

PBH, especially in those with severe, recurrent hypoglycemia, can lead to adverse outcomes, including impaired cognitive function and even mortality, warranting prompt treatment and implementation of preventive strategies to reduce future risk. Frequent hypoglycemia also increases the risk of developing hypoglycemia unawareness [134]. Glucagon is an emergency treatment for severe hypoglycemia in insulin-treated diabetes. The American Diabetes Association recommends glucagon as a rescue treatment that can be administered by healthcare providers, as well as family members or other caregivers, for individuals at high risk of hypoglycemia [135]. However, there are insufficient studies evaluating the role of glucagon treatment in the acute treatment of hypoglycemia, specifically in PBH patients. An exploratory observational study by Lobato et al has attempted to understand the entero-pancreatic hormone dynamics in post-RYGB PBH and helped identify the potential role of glucagon in preventing hypoglycemia. The study's findings show that an earlier postprandial (15 minutes after the meal) glucagon excursion prior to the insulin peak may help increase glucose levels at nadir and prevent hypoglycemia [34]. A phase 2 placebo-controlled double-blind study conducted in 2 phases—a crossover training phase and a real-world phase—evaluated the efficacy of a ready-to-use mini-dose liquid glucagon (XP-5387) in normalizing glycemic levels in PBH patients experiencing acute hypoglycemic events. Euglycemia was restored from baseline self-monitored blood glucose value of <70 mg/dL without glucose tablets in 78.6% of XP-5387 dosing events vs 64.5% of placebo dosing events [136]. Based on the findings of these new studies and recommendations by the Society for Endocrinology 2024, and given the long-term and short-term consequences of acute hypoglycemic episodes, glucagon may be considered for certain PBH patients at risk of severe and frequent hypoglycemia [23, 75].

##### Calcium channel blockers

Calcium channel blockers (CCBs), such as nifedipine and verapamil, block insulin secretion by inhibiting the voltage-gated calcium channels on pancreatic beta cells [112]. A few case reports have shown successful management of PBH with dietary modifications plus a CCB, either alone or in combination with acarbose [137-140]. On the other hand, a study by Ohrstrom et al did not find any effect of verapamil on fasting or postprandial glucose metabolism [28, 116]. Thus, further research is needed to establish their efficacy and optimal use in PBH management.

#### Surgical management of PBH: a last resort option

Due to the potential risks and complications associated with surgical procedures, they should be reserved for severe cases that are either medically refractory (incomplete resolution of symptoms despite an adequate trial period of at least 12 weeks of all appropriate dietary measures and all lines of pharmacological interventions, with confirmed treatment adherence) or complicated by hypoglycemia unawareness [136, 141-145]. Nonsurgical management strategies, including dietary modifications and pharmacotherapy, should be exhausted before contemplating surgery. Surgical interventions should be carefully considered on a case-by-case basis, following a thorough evaluation of their potential risks and benefits. To mitigate symptoms in severe, treatment-refractory PBH, gastrostomy tube placement in the remnant stomach may be considered to provide controlled enteral nutrition, replacing oral feeding [146]. Other surgical interventions, such as reversal of RYGB, should be considered as a last resort.

### Considerations During Pregnancy

Studies show that more than half of pregnant women experience postprandial hypoglycemia following gastric bypass [48, 147]. After MBS, pregnancy in women should be considered high-risk, with additional vigilance in those with PBH due to the potential adverse effects of recurrent hypoglycemia on both the mother and fetus [50, 148, 149]. The increased risk for potential complications underscores the importance of referring pregnant PBH patients to an experienced endocrinologist who can closely monitor and manage their glucose levels throughout pregnancy to ensure maternal well-being and improved fetal outcomes. Moreover, managing PBH during pregnancy calls for a multidisciplinary team approach that integrates the expertise of different specialties to help mitigate the risk for both the mother and baby.

There are limited pharmacotherapies available for the treatment of nonpregnant PBH patients, and pregnancy further limits options due to the potential risks of fetal harm associated with medications. Thus, dietary modification is the primary strategy for PBH management during pregnancy. Educating patients about their diagnosis and the significance of following the recommended dietary plan can empower them to achieve glycemic control and potentially eliminate the need for medications [148].

Acarbose can be considered as the “least hazardous pharmacological option for PBH management in pregnancy.” [150] Large studies on the efficacy of acarbose in pregnant PBH patients are lacking, with the evidence limited to a few published case reports. Narayanan and Syed report the successful treatment of a 25-year-old pregnant PBH patient with acarbose [150]. A more recent abstract published by Melendez-Rivera and Parikh describes the use of acarbose in a 37-year-old pregnant PBH patient. It was less effective in this case, as the patient needed multiple hospital admissions for severe hypoglycemic episodes despite being on acarbose. Octreotide also had to be initiated during one of her hospital admissions, as the potential benefits seemed to outweigh the risks. The patient's symptoms improved in the third trimester [151]. This case highlights: (i) the challenges of managing PBH beyond dietary modification during pregnancy due to the lack of safe and effective therapeutic options; (ii) the need to balance maternal glycemic control with fetal safety while selecting treatment; and (iii) the importance of personalizing treatment plans by carefully evaluating the risks and benefits of the available treatment modalities. However, based on the limited evidence available, acarbose may be considered in the treatment of PBH during pregnancy after securing patients’ informed consent to initiate or continue its administration.

OGTT is routinely performed to detect hyperglycemia during pregnancy. However, studies show significant alterations in plasma glucose levels following an oral glucose load in post-bariatric pregnant individuals, raising questions about the diagnostic utility of the OGTT in this cohort [147, 149, 152, 153]. Due to the potential risk of hypoglycemia following an OGTT, alternative strategies to detect hyperglycemia should be considered in this population [147].

### Long-Term PBH Management and Monitoring

Routine follow-up and close monitoring of patients after initiating treatment—whether through dietary modifications or pharmacotherapy—is crucial. This will enable physicians to assess treatment efficacy, detect adverse effects, promote patient compliance, and promptly adjust treatment plans if needed.

Unlike point-of-care CBG measurements, a regulatory agency-approved CGM system can help evaluate real-time and continuous glucose levels, allowing prompt detection of hypoglycemia [154]. Continuous monitoring capability of CGM can serve as a practical tool for assessing long-term treatment efficacy and guiding decision-making. Although studies have not specifically examined the effect of CGM on treatment adherence, visualizing the immediate consequences of noncompliance may help improve patients’ adherence to medical advice, thereby enhancing overall outcomes.

“Hypoglycemia unawareness” is a condition occurring as a result of repeated episodes of hypoglycemia and in which individuals fail to experience its typical symptoms, leading to an increased risk of severe hypoglycemic events without warning [28, 59, 155]. Hypoglycemia unawareness has been shown to be prevalent in the PBH patient cohort, further reinforcing the need for effective, long-term monitoring strategies [156]. Cummings et al conducted an open-label, nonrandomized study by evaluating the CGM data from 22 participants diagnosed with PBH in 2 phases: first, a masked monitoring phase, in which participants could not access their sensor glucose data and alarms, followed by an unmasked monitoring phase, in which participants had access to sensor glucose data and alarms (for low or rapidly decreasing glucose levels). The findings showed that access to the real-time CGM data and alarms during the unmasked phase enabled the participants to monitor glycemic trends, including impending hypoglycemic episodes, leading to a clinically meaningful improvement in the time spent in the target glycemic range (between 3.9 and 10 mmol/L) [157, 158]. Based on the evidence that is slowly accruing on the utility of CGM, and depending on its cost and availability, a regulatory agency-approved CGM system may be considered for monitoring patients who may be experiencing hypoglycemia unawareness [155-157, 159].

Fear of hypoglycemia has been shown to impact the psychological well-being of diabetes patients adversely. It can lead to heightened anxiety, affecting sleep and impairing day-to-day domestic and social activities, and can also encourage excessive snacking [160-162]. An ongoing fear of hypoglycemia can thus contribute to poor QoL and possibly weight regain, and it is a crucial factor to consider while managing PBH patients as well. Involving a psychologist with expertise in obesity medicine as a part of a multidisciplinary team can help address the psychological aspects of PBH whenever clinically indicated and ensure a more holistic approach to its management.

Modern-day technologies such as mobile applications, remote education, and online teleconsultation can serve as valuable tools in the long-term care of PBH patients. These tools can enable more regular monitoring, improve communication, and provide patients with a platform for ongoing education and support. However, these technologies are not utilized optimally in the Middle East region [163, 164]. Integrating them into PBH management could enhance postintervention care, reduce long-term complications, improve treatment outcomes, and empower patients to better understand and manage their condition.

### Unmet Needs and Emerging Trends in PBH Management

Glycemic variability refers to changes in blood glucose levels—hyperglycemic spikes and hypoglycemic episodes—throughout the day, as well as fluctuations that recur at the same time on different days. Increased glycemic variability has been identified as an independent contributor to diabetes-related complications [165]. Increased glycemic variability has also been observed in post-bariatric individuals, possibly contributing to an increased risk of hypoglycemia [166-169]. A prospective, longitudinal, observational study by Nilsen et al compared the 24-hour interstitial glucose levels, glycemic variability, hypoglycemia incidence, and dietary intake in females without diabetes before and after laparoscopic RYGB and SG. Glycemic variability was increased at the 6-month and 12-month postoperative time points, with the increase being more pronounced in the RYGB group than the SG group [167]. Glycemic variability data provided by systems like the flash glucose monitor are being analyzed for their potential to improve PBH diagnosis and management. In the future, when extended to the clinical setting, this capability, coupled with a detailed food and symptom diary, could help stratify post-bariatric individuals based on their risk for PBH. It could also facilitate the development of personalized treatment and monitoring strategies to manage PBH [170].

Although therapeutic options for PBH beyond dietary modifications are limited, emerging therapies like mizagliflozin, a selective SGLT-1 inhibitor that can reduce intestinal glucose absorption, offer hope [171, 172]. In a phase 2 randomized study, mizagliflozin has been shown to increase glucose nadir and reduce postprandial glucose and insulin peaks after an MMTT [172]. A brief overview of other emerging therapies under investigation is shown in Table 9 [172-177]. Also, conducting head-to-head randomized controlled trials comparing the efficacy of existing pharmacotherapies, such as pasireotide and octreotide, in PBH can help guide treatment choices.

Additionally, in the context of unmet needs, we acknowledge that concerning the widespread observance of Ramadan in the GCC region, current data are insufficient to direct detailed guidance in this regard, underscoring the urgent need for robust studies in this research area in the PBH cohort.

### Practical Considerations in PBH Management

MBS candidates should be informed about potential postoperative challenges and the nutritional recommendations they must follow. This proactive counseling may help raise awareness about complications like PBH and allow them to participate actively in its management. Physicians should evaluate candidates’ readiness to adopt behavioral modifications, such as any dietary and lifestyle adjustments, and actively counsel them if needed to ensure long-term adherence to treatment plans and improved outcomes.

Secondary and tertiary healthcare institutions in the Gulf region should prioritize the recruitment and training of nutritionists who can provide personalized guidance on dietary modifications to PBH patients. This expertise can help patients navigate the complex nutritional challenges following MBS.

Hypoglycemia unawareness in those diagnosed with PBH can cause blood glucose levels to drop without warning. Thus, it can be challenging for PBH patients to take timely corrective actions to avoid endangering themselves and those around them. This condition increases the likelihood of accidents and injuries, particularly while driving or operating heavy machinery [23, 178]. Policymakers should implement regulations and licensing requirements to ensure the safety of PBH patients who may be at risk for hypoglycemia unawareness. Requiring mandatory CGM with a regulatory agency-approved system and alarm features or algorithms to predict hypoglycemia before it occurs for such individuals while obtaining a driving license or employment in high-risk jobs can help mitigate risks to some extent. Balancing public safety with the medical and employment needs of PBH patients is crucial.

## Discussion

Obesity has emerged as a significant public health challenge in the MENA region, with prevalence rates in some of the countries being among the highest globally [6]. This increased obesity prevalence has led to a steep rise in the number of bariatric surgeries performed to manage severe obesity and its complications. Moreover, the 2022 ASMBS/IFSO guidelines now recommend MBS in individuals with class I obesity (BMI 30-34.9 kg/m^2^) who do not respond to nonsurgical treatments [16, 179]. PBH, a complication of MBS characterized by postprandial hypoglycemic episodes, poses diagnostic and therapeutic challenges due to its heterogeneous presentations, diverse pathophysiology, inconsistent diagnostic criteria, and limited therapeutic options. Substantial variability in diagnosing and managing PBH complicates clinical decision-making and delays timely intervention, increasing the risk of long-term complications. Developing region-specific consensus on the management of PBH that accounts for the unique social, cultural, dietary, and lifestyle factors, as well as healthcare infrastructure, expertise, and availability of diagnostic tools and treatments, is essential for ensuring uniformity in care across the Middle East region.

Through a collaborative process, a panel representing all the GCC countries has developed a set of consensus statements presented in this paper with the goal of standardizing the region's diagnostic and treatment pathway for PBH. These statements cover the entire spectrum of PBH management, which includes initial screening, diagnostic confirmation, dietary and pharmacological treatment, special considerations during pregnancy, long-term management and monitoring, unmet needs and emerging trends, and practical considerations. However, a limitation of this consensus is that, given the scarcity of studies in PBH, greater emphasis had to be placed on expert discussion based on clinical judgment and collective experience rather than on empirical evidence.

We also acknowledge that while these consensus statements represent a significant step forward in standardizing PBH management in the GCC region, universally accepted diagnostic criteria and treatment protocols are needed to further optimize PBH management and improve patient outcomes. Although developed from a GCC-specific perspective, these statements may broadly apply to other regions with comparable healthcare systems and clinical practice settings.

## Conclusions

PBH management presents significant challenges due to heterogeneity in diagnostic criteria and tools, treatment approaches, and monitoring strategies. Clinicians have limited effective pharmacological options in patients unresponsive to dietary management, further complicating PBH management. Addressing these gaps calls for multidisciplinary collaboration to standardize the diagnostic and treatment protocols and additional research efforts to identify novel therapies specifically for PBH.

## Data Availability

Original data generated and analyzed during this study are included in this published article or in the data repositories listed in References.

## Abbreviations

ASMBS: American Society for Metabolic and Bariatric Surgery
BMI: body mass index
CBG: capillary blood glucose
CCB: calcium channel blocker
CGM: continuous glucose monitoring
FDA: Food and Drug Administration
FGF: fibroblast growth factor
GCC: Gulf Cooperation Council
GIP: glucose-dependent insulinotropic polypeptide
GLP-1: glucagon-like peptide 1
GLP-1 RA: glucagon-like peptide 1 receptor agonist
GOSEEN: Obesity Group of the Spanish Society of Endocrinology and Nutrition
IFSO: International Federation for the Surgery of Obesity and Metabolic Disorders
IL-: interleukin
IM: intramuscular
LAR: long-acting repeatable
MBS: metabolic and bariatric surgery
MENA: Middle East and North Africa
MMTT: mixed meal tolerance test
OGTT: oral glucose tolerance test
PBH: post-bariatric hypoglycemia
QoL: quality of life
RYGB: Roux-en-Y gastric bypass
SC: subcutaneous
SG: sleeve gastrectomy
SGLT: sodium-glucose cotransporter
SSA: somatostatin analogue
UAE: United Arab Emirates
